# Peritoneal dialysis-associated peritonitis caused by *Exophiala spinifera*: A case report and review of literature

**DOI:** 10.1016/j.mmcr.2021.12.006

**Published:** 2022-01-28

**Authors:** Pongsagorn Jabgratog, Tamonwan Chamroensakchai, Talerngsak Kanjanabuch, Jureeporn Ampaipun, Nisa Thongbor, Vedprakash G. Hurdeal, Kevin D. Hyde

**Affiliations:** a Trakan Phuet Phon Hospital, Ubon Ratchathanee, Thailand; b Center of Excellence in Kidney Metabolic Disorders, Faculty of Medicine, Bangkok, Thailand; c Division of Nephrology, Department of Medicine, Faculty of Medicine, Chulalongkorn University, Bangkok, Thailand; d CAPD Excellent Center, King Chulalongkorn Memorial Hospital, Bangkok, Thailand; e Sunpasitthiprasong Hospital, Ubon Ratchathanee, Thailand; f Center of Excellence in Fungal Research, Mae Fah Luang University, Chiang Rai, 57100, Thailand; g School of Science, Mae Fah Luang University, Chiang Rai, 57100, Thailand

**Keywords:** *Exophiala spinifera*, Peritoneal dialysis, Peritonitis, Phaeohyphomycosis, Cutaneous infection, FP, Fungal peritonitis, PD, Peritoneal dialysis, HD, Hemodialysis, KOH, Potassium hydroxide, SDA, Sabouraud dextrose agar, EUCAST, The European Committee on Antimicrobial Susceptibility Testing, MIC, Minimal inhibitory concentration, DR, Diabetic retinopathy, ISPD, International Society for Peritoneal Dialysis

## Abstract

*Exophiala spinifera* is a black ascomycetous yeast and is responsible for phaeohyphomycosis. We provide the first case report of peritoneal dialysis (PD)-associated peritonitis in a female patient with progressive impairment of visual capacity. The infection was caused by a cutaneous infection of her hands. The patient responded well with PD catheter removal and 2-week antifungal medication. This case emphasizes the importance of hand hygiene and regular eye evaluation in preventing environment-bound infection in patients on PD. 2012 Elsevier Ltd. All rights reserved.

## 1 Introduction

Fungal peritonitis (FP) is rare but is associated with high morbidity and mortality rates [1]. *Exophiala* species are black yeast-like fungi found worldwide, inhabiting soil, wood, and dead plant matters. These fungi can cause chronic, localized infections of the cutaneous and subcutaneous tissues and cornea, known as phaeohyphomycosis [2]. In addition, the infection sometimes invades bone tissue causing bone necrosis [[3], [4], [5]]. Phaeohyphomycosis is not specific to *Exophiala* but refers to infections from dematiaceous or pigmented filamentous fungi which contain melanin in their cell walls, such as *Bipolaris, Cladophialophora, Cladosporium, Fonsecaea, Phialophora, Ochronosis, Rhinocladiella*, and *Wangiella* [6].

Systemic fungal infections are rare in people with normal host immunity but can be observed in the lungs, lymph nodes, and central nervous system in immunocompromised hosts [4,7]. There are eight case reports of *Exophiala* peritonitis in the literature caused by *E. dermatitidis, E. jeanselmei,* or *E. xenobiotica* [[8], [9], [10], [11], [12], [13], [14], [15]]. Here we provide the first report of peritoneal dialysis (PD)-associated peritonitis caused by *E. spinifera*, and review of the literature on *Exophiala* peritonitis in PD patients.

## 2 Case

**Fig. 1:**
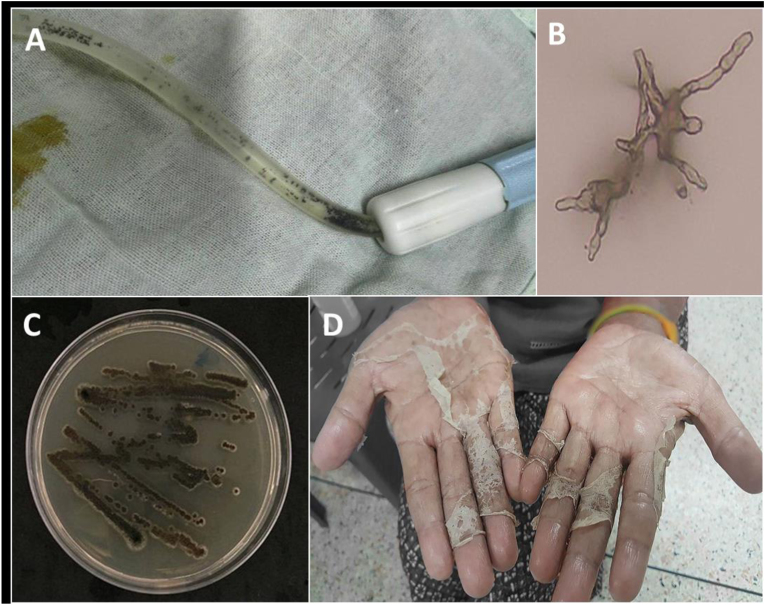
Numerous black granules distributed inside the peritoneal catheter (A) with dark brown septate hyphae with branches in 45° in the microscopic exam (KOH; 100x) (B). The dark colony on Sabouraud-Dextrose agar was demonstrated after ten days of incubation at 25 °C (C). In addition, many black specks and dirt were observed on her desquamated skin of both hands (D). (For interpretation of the references to colour in this figure legend, the reader is referred to the Web version of this article.)

A 52-year-old Thai female farmer with diabetic kidney failure and long-standing hypertension had been on PD (1.5% dextrose concentration x 4 exchanges/day) for over a year without infectious complications, at Trakan Phuet Phon Hospital, a community hospital in the northeastern part of Thailand. She was presented with cloudy effluent and dark granules inside her PD catheter without systemic signs of infection on day 0 (Fig. 1A). The patient was referred to Sunpasitthiprasong Hospital, a tertiary hospital, on the same day. The dialysate cell counts on day 0 showed numerous erythrocytes and leukocytes 26,000 cells/mm^3^ with 78% lymphocytes and 22% neutrophils. Her initial diagnosis was fungal peritonitis with intraluminal PD catheter colonization. A 2-week course of intravenous amphotericin B was immediately prescribed at 1 mg/kg/day, the PD catheter was removed, and hemodialysis (HD) was commenced on day +1.

**Fig. 2:**
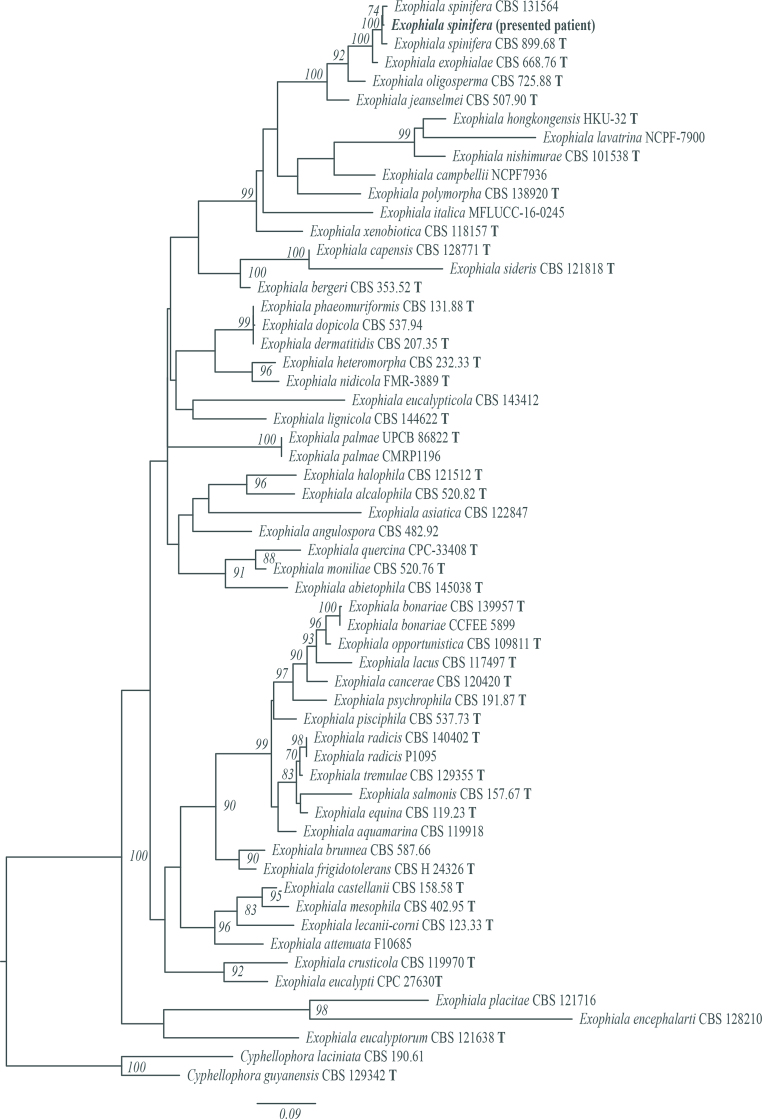
The phylogenetic tree of the identified organism is consistent with *E. spinifera*.

The removed PD catheter and used PD bag were submitted to a central laboratory for microorganism identification. Direct microscopic examination with 20% potassium hydroxide (KOH) mount from the catheter revealed dark brown branching septate hyphae (Fig. 1B). Black colonies grew on Sabouraud dextrose agar (SDA) after seven days of incubation of both specimens. As the colony matured, the surface of colonies was gradually covered with a gray velvety mycelium (Fig. 1C). The taxon was identified as *Exophiala* spp. using API20c AUX kit (bioM'erieux, Marcy l’Etoile, France) based on biochemical reactions. Later on, the species was confirmed to be *E. spinifera* using the universal fungal primers ITS1/ITS4 to amplify the ITS gene region. Blast search against the nucleotide database in GenBank showed a 100% (1031/1031) identity to *E. spinifera* (accession number MN410626.1) (First BASE Laboratories, Singapore Science Park II, Singapore). The phylogenetic tree is shown in Fig. 2. Using the European Committee on Antimicrobial Susceptibility Testing (EUCAST) Antifungal Clinical Breakpoint referenced to a microbroth dilution value of *Aspergillus* spp. [16], the pathogen was resistant to itraconazole (4μg/mL), fluconazole (>64μg/mL), and caspofungin (>64μg/mL) and susceptible to amphotericin B and voriconazole with minimal inhibitory concentrations (MICs) of 0.25 and 1.0 μg/mL, respectively. The patient responded well to the antifungal medication, as shown by the modified Edmonton Symptom Assessment System score decreasing from 19 at the onset of infection to zero. However, the patient had a decreased visual acuity due to proliferative diabetic retinopathy (DR), and the caregiver was unavailable. Therefore, the physician permanently transferred the patient to HD.

To define the etiology of the infection, the physician performed a root-cause analysis. The patient had DR in both eyes, which significantly affected her visual acuity at the initiation of PD. However, the patient could perform PD bag exchange by herself without a caregiver. The level of visual impairment became worse prior to the episode of infection; however, the patient did not report this information to her PD care team and continued to perform the exchange by herself. PD bag exchange and aseptic techniques were properly conducted, but reluctance was detected during the bag exchange steps. However, many black specks and dirt were observed in desquamated skin on her palms (Fig. 1D). Therefore, fungal colonization on the peel-off skin was suspected. Skin flakes were cultured, and the fungus recovered was the same species as the pathogen causing the peritonitis. Therefore, it is concluded that palm colonization by *E. spinifera* might be the most likely source of the infections. The skin infection was eradicated with a month course of topical ketoconazole and meticulous hand hygiene practice.

## 3 Discussion

*Exophiala* species are dematiaceous fungi classified within Herpotrichiellaceae (Eurotiomycetes/Chaetothyriales) which have a well-established etiology of phaeohyphomycosis [17]. *Exophiala dermatitidis* and *E. jeanselmei* are relatively common pathogens. This case represents the first case of PD-associated peritonitis caused by *E. spinifera*. Eight cases of *Exophiala* peritonitis have been reported in PD patients since 1983 (Table 1). There are three males (38%) with an average age of 43 (2–66) years, and two cases presenting with concomitant intraluminal colonization of *Exophiala*.

**Table 1:** Demographic and treatment outcome of *Exophiala* peritonitis.

No	Gender	Ages (years)	Identified organism	Diagnostic method	Underlying condition	Associated finding	Antifungal therapy	Catheter removal	Outcome	Reference
1	M	42	*E. jeanselmei*	Culture	NA	No	FC and AMB	No	Died	Kerr 1983 [8]
2	M	39	*E. dermatitidis*	Culture	GN	No	FCZ	Yes	Survived	Lye 1993 [9]
3	F	19	*E. jeanselmei*	Culture	Congenital	Cath.	AMB	Yes	Survived	Agarwal 1993 [10]
4	F	57	*E. jeanselmei*	Culture	AIN	Cath.	FC and FCZ	Yes	Survived	Remon 1996 [11]
5	F	53	*E. dermatitidis*	Culture	NA	No	FCZ	Yes	Survived	Vlassopoulos 2001 [12]
6	F	55	*E. dermatitidis*	Culture	PKD	No	AMB	Yes	Survived	Greig 2003 [13]
7	M	66	*E. xenobiotica*	Seq.	DM, HT, CAD	ESI	AMB	Yes	Survived	Lau 2003 [14]
8	F	2	*E. dermatitidis*	Culture, Seg.	None	No	VRC	Yes	Survived	Pinheiro 2019 [15]
9	F	52	*E. spinifera*	Seq.	DM, HT	Cath.	AMB	Yes	Survived	The present case

**Abbreviations:** AIN, acute interstitial nephritis; AMB, amphotericin B; cath, catheter colonization; CAD, coronary artery disease; DM, diabetes mellitus; FCZ; fluconazole; FC, flucytosine; GN, glomerulonephritis; HT, hypertension; HIV, human immunodeficiency viruses; NA, not available; PKD, polycystic kidney disease; Seq, gene sequencing; UK, United Kingdom; USA, United States of America; VRC, voriconazole.

Since no specific recommendations are made over this type of fungal pathogen [18], the 2016 International Society for Peritoneal Dialysis (ISPD) Peritonitis Guidelines [19] only recommend that “treatment with an appropriate antifungal agent be continued for at least 2 weeks after catheter removal.” In all cases of *Exophiala* peritonitis with PD catheter removal, favorable outcomes with complete/partial resolution were described after complete treatment (7/7, 100%), while only one case with catheter retained in-situ was resilient [8]. Table 1 indicated that amphotericin B is the most common antifungal agent used to treat *Exophiala* peritonitis, resulting in complete remission in 4 out of 5 cases [10,13,14]. The second typical therapy is fluconazole, with 100% effectiveness in eliminating the infection [9,11,12]. Despite lacking a standard treatment of *E. spinifera* peritonitis [18] and its rarity, amphotericin B administration for two weeks with early catheter removal provided an excellent clinical outcome in accordance with the in-vitro MIC results.

Although the source of the infection is inconclusive in the presented case, superficial fungal infection of the patient's desquamated palm skin is the most probable explanation since the same pathogen was found on the patient's hands. Impeccable hand hygiene is crucial during PD bag exchange in preventing peritonitis. Any visibly dirty hands require washing with antiseptic soap. Patients, healthcare providers, and patient caregivers should be aware of the importance of proper hand hygiene protocols [20]. If the patient's hand is infected, treatment should be started. Self-performing bag exchanges should be withheld, or sterile gloves should be used. Blindness is relatively contraindicated to start PD; however, the cut-off level of visual impairment has not yet been discussed in the PD society. Notably, a decline in the patient's visual acuity over time should be considered, and periodic assessment should be incorporated as a part of routine PD care, particularly in patients with diabetic retinopathy and extreme age [21]. The skin infection should receive attention. In conclusion, we reported the first case of fungal peritonitis caused by *E. spinifera* associated with cutaneous infection of the patient's hands. Thus, this case raises awareness of the decline in patient visual acuity over time and reemphasizes the importance of hand hygiene in preventing peritonitis.

## Competing interests

TK has received consultancy fees from VISTERRA as a country investigator and current recipient of the National Research Council of Thailand and received speaking honoraria from Astra Zeneca and Baxter Healthcare. All other authors have no financial conflicts of interest to declare.

## Funding for your research

This study was supported by the 10.13039/501100017170Thailand Science Research and Innovation Fund 10.13039/501100002873Chulalongkorn University CU_FRB65_hea (19)_026_30_07, 10.13039/501100002873Chulalongkorn University, Bangkok, Thailand and the 10.13039/501100004704National Research Council of Thailand (156/2560), Thailand.

## Consent

The patient provided written informed consent to this case's publication. The consent form is available for review by the Editor of this journal.
